# Serum Levels of LL-37 and Inflammatory Cytokines in Plaque and Guttate Psoriasis

**DOI:** 10.1155/2014/268257

**Published:** 2014-08-14

**Authors:** Young Ji Hwang, Ho Jung Jung, Min Jung Kim, Nam Kyung Roh, Jae Wook Jung, Yang Won Lee, Yong Beom Choe, Kyu Joong Ahn

**Affiliations:** Department of Dermatology, Konkuk University School of Medicine, 120-1 Neungdong-ro, Gwangjin-gu, Seoul 143-729, Republic of Korea

## Abstract

Psoriasis is a chronic inflammatory skin disease. It is assumed that the plaque phenotype of psoriasis is associated with T helper (Th) 1 immune response activation, while the guttate phenotype is associated with the Th17 immune response. Previous investigations of differences in the serum levels of cytokines relative to the clinical psoriatic phenotype have yielded conflicting results. This study compared the levels of circulating inflammatory cytokines and LL-37 relative to the morphological phenotype in patients with psoriasis. Seventy-four age-matched patients with psoriasis (32 with guttate psoriasis and 42 with plaque psoriasis) and 12 healthy controls were included. A multiplex cytokine assay and enzyme-linked immunosorbent assay were used to measure levels of Th1- and Th17-derived cytokines and LL-37, respectively. Circulating levels of interferon- (IFN)-*γ*, interleukin- (IL)-1RA, IL-2, and IL-23, and LL-37 were significantly higher in patients with psoriasis than in healthy controls. However, the serum levels of inflammatory cytokines (IL-7, IL-22, and IL-23) and LL-37 did not differ significantly between the guttate and plaque phenotypes of psoriasis. There was a positive correlation between serum inflammatory cytokine levels and the Psoriasis Area and Severity Index score. The findings of this study suggest that the serum levels of inflammatory cytokines reflect the disease activity rather than determine the morphological phenotype.

## 1. Introduction

Psoriasis is a chronic inflammatory skin disease with characteristic histological changes, including abnormal epidermal proliferation and a cellular infiltrate composed of neutrophils and T cells [1]. Psoriasis was originally classified as a T helper (Th) 1 disease because cytokines involved in the Th1 pathway, such as interferon- (IFN-) *γ*, interleukin- (IL-) 2, and IL-12, are elevated in lesional skin and peripheral blood [2–4], and the success of classical treatments is related to the result of redirecting a Th1 response towards a Th2 response [5]. Recent evidence suggests that a newly recognized subset of Th cells (Th17 cells, characterized by IL-17-producing CD4+ effector T cells) plays an important role in the pathogenesis of psoriasis [6]. Th17 cells differentiate from naïve CD4+ T cells when stimulated by IL-1*β*, IL-6, and IL-23 and can produce IL-17, IL-21, and IL-22 [7].

Recent studies also suggest that dendritic cells (DCs) and altered expression of antimicrobial peptides play a role in the pathogenesis of psoriasis. Plasmacytoid DCs are activated through a cathelicidin LL-37 and DNA complex in a toll-like-receptor-dependent manner [8]. This activation induces increased production of type I IFN, leading to myeloid DC activation and consequently leading to Th1/Th17 differentiation and keratinocyte activation [9]. This subsequently induces the expression of various cytokines [10, 11]. Both expression on lesional skin and systemic levels of LL-37 increase as a result and a correlation was found between LL-37 expression and proinflammatory cytokines in patients with psoriasis [12]. However, there has been a relative dearth of data regarding the circulating levels of LL-37 and the correlations with specific serum cytokines.

Psoriasis is classified into 5 morphological subtypes [13], although it is also thought that the phenotypes may transform into other clinical forms of the disease [14]. It has recently been suggested that IL-12/IFN-*γ* or IL-23/IL-17 signaling can influence the determination of clinical phenotypes. For example, the plaque and guttate types of psoriasis are thought to be related to Th1 cytokines and Th17 immune responses, respectively. However, few studies have attempted to determine the correlations of biochemical markers with clinical phenotypes.

The present study compared differences in the serum levels of circulating Th1 and Th17 cytokines between plaque and guttate psoriasis and investigated the correlation between disease activity and serum levels of inflammatory cytokines. In addition, serum LL-37 levels in patients with psoriasis were compared with those in healthy controls, and the correlations between serum levels of LL-37 and inflammatory cytokines were analyzed.

## 2. Methods

### 2.1. Participants

Seventy-four patients with psoriasis (44 males and 30 females) and 12 healthy controls without psoriasis and without any family history of psoriasis were included in this study (Table 1). The controls were age- and sex-matched; accordingly, the age and sex distributions did not differ between the patient and control groups (*P* > 0.05). Psoriasis was diagnosed clinically and histopathologically and the following major inclusion criteria were implemented: no local or systemic treatment for at least 4 weeks prior to entering the study, no significant infection or immune suppression, and no history of specific medical diagnoses with renal, hepatic, cardiovascular, pulmonary, rheumatic, or endocrine involvement. Patients with erythrodermic, pustular, or palmoplantar-specific forms of psoriasis were excluded, as were those with psoriatic arthritis. The severity of the disease was evaluated by using the Psoriasis Area and Severity Index (PASI) score. The patients were divided into either the guttate or the plaque group according to their morphological psoriasis phenotype at the time of admission. Guttate psoriasis was defined as acute onset or reactivation of scattered, small plaque lesions of <1 cm in diameter, while patients with plaque psoriasis exhibited nummular and large plaques (≥1 lesion with a long-axis diameter of >5 cm). A total of 74 patients were assigned to either the guttate (*n* = 32) or plaque (*n* = 42) psoriasis group. The mean duration of disease and age did not differ between the 2 psoriasis groups (*P* > 0.05) as they had been matched for age and disease duration.

This study was conducted in accordance with the guidelines from the Helsinki Conference (52nd World Medical Association General Assembly, Edinburgh, United Kingdom, October 2000) and Korean Good Clinical Practice, with the participants' rights and safety taking precedence. Approval from the institutional review board was obtained. All participants were provided with detailed information about the study's purpose, methods, and expected results, after which the patients' informed consent to participate was obtained prior to screening.

### 2.2. Collection and Preparation of Blood Samples

Venous blood samples (5–10 mL) were collected into vacuum tubes under sterile conditions from both the patient and control groups. Serum was obtained by spinning the samples in a centrifuge and then immediately frozen at −70°C and stored until required for analysis.

### 2.3. Assays

Multiple cytokine analysis was performed by using xMAP technology (Luminex 200, Luminex, Austin, TX, USA) to measure serum IFN-*γ*, IL-1 receptor antagonist (IL-1RA), IL-2, IL-12(p40), IL-17A, IL-22, and IL-23. The Milliplex MAP multiplex assay was conducted in a 96-well microplate format according to the manufacturer's recommendations (Millipore, Billerica, MA, USA). Briefly, each of the bead solutions was transferred into a mixing vial and adjusted to a volume of 3 mL with bead diluents. Internal controls and standards, ranging from 0 to 10 000 pg/mL for each cytokine, were included with every assay. Following the addition of sera and beads, the resulting mixture was incubated overnight at 4°C. Detection antibodies and streptavidin-phycoerythrin were then added sequentially at room temperature for 30 minutes, and the plate was analyzed with the Luminex 200 system.

Serum cathelicidin LL-37 levels were measured by performing an enzyme-linked immunosorbent assay with commercially available kits (Hycult Biotech, Plymouth Meeting, PA, USA). The minimum detectable concentration of serum LL-37 when using this assay was 0.15 ng/mL.

### 2.4. Statistical Analysis

All data were analyzed by using the Statistical Package for the Social Sciences (SPSS) 17.0 software for Windows (SPSS, Chicago, IL, USA). The Mann-Whitney *U* test was used to compare mean values between groups and correlation analysis was performed by using the Pearson correlation test. Differences were considered to be statistically significant when *P* < 0.05.

## 3. Results

### 3.1. Comparison of Serum Levels of Cytokines and LL-37

Circulating levels of cytokines and LL-37 were analyzed in both the patient and control groups (Table 2). Serum levels of IL-1RA, IL-2, IL-23, IFN-*γ*, and LL-37 were elevated in patients with psoriasis compared with the controls. Although the serum IL-12(p40), IL-17A, and IL-22 levels also appeared to be elevated compared with the healthy control group, the difference was not statistically significant in the experimental setting of our study. Furthermore, the serum levels of all the tested cytokines and LL-37 did not differ significantly between the guttate and plaque psoriasis groups (Table 3).

### 3.2. Correlations between Serum Levels of Cytokines and LL-37 and PASI

**Figure 1:**
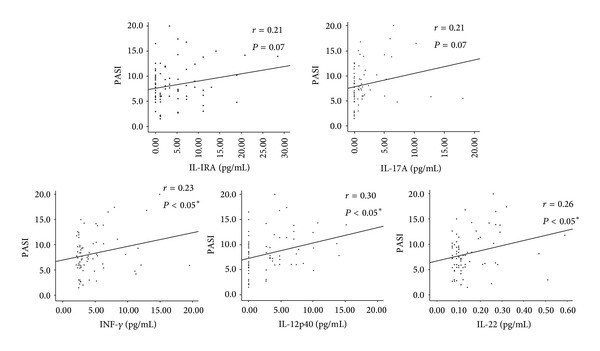
Correlations between serum levels of interleukin- (IL-) 1 receptor antagonist (RA), IL-12 (p40), IL-17A, IL-22, and interferon (IFN)-*γ* and Psoriasis Area and Severity Index (PASI) scores in patients with psoriasis. The Pearson correlation test and linear regression were used to calculate the linear regression lines.

The serum levels of 7 inflammatory cytokines and LL-37 in the 74 patients with psoriasis were compared with disease severity (as defined by PASI score) in order to establish the presence of any correlations. IL-12(p40), IL-22, and IFN-*γ* were significantly and positively correlated with PASI in all patients with psoriasis (Table 4, Figure 1). Although not statistically significant, there was a tendency for both IL-1RA and IL-17A to be positively correlated with PASI (Figure 1).

### 3.3. Correlations between Serum Levels of Individual Cytokines and LL-37

The correlations between the serum levels of LL-37 and individual cytokines are presented in Table 5. Serum LL-37 levels were significantly correlated with serum IL-22 and IFN-*γ* levels in patients with psoriasis. Although the correlations between serum IL-17A, IL-23, IL-1RA, and LL-37 levels were statistically nonsignificant, there appeared to be a trend towards an overall positive correlation (*P* = 0.09, *P* = 0.15, and *P* = 0.18, resp.). These findings suggest the presence of interplay between LL-37 in innate immunity and the cytokines involved in the Th1 and Th17 immune responses in psoriasis.

## 4. Discussion

Psoriasis is a chronic inflammatory skin disease involving the induction of Th1 and Th17 cell responses and the aberrant expression of proinflammatory cytokines [15]. The findings of the present study confirm previous reports [16, 17] that the serum levels of most of the Th1- and Th17-related cytokines are elevated in the serum of patients with psoriasis.

The patients included in the present study were divided into the following 2 morphological phenotypes of psoriasis: guttate and plaque. Comparing the levels of serum inflammatory cytokines between these psoriasis groups revealed no statistically significant differences. These results suggest that morphological phenotype is not determined by specific activities of either the Th1 or Th17 pathway. A few studies have compared the serum cytokine levels among psoriasis subgroups, but their results were conflicting and did not definitively determine whether there is a significant relationship between the specific cytokines and phenotypes [18–20]. The present study is the first to compare the serum levels of inflammatory cytokines in different morphological phenotypes of psoriasis with similar disease duration and may provide a partial explanation for the conflicting results regarding cytokine levels previously reported. Thus, it seems that both Th1 and Th17 pathways are associated with the pathogenesis of psoriasis.

This study found significant correlations between the serum levels of IFN-*γ*, IL-12(p40), and IL-22 and patients' PASI scores. Combined with results of previous studies [3, 21, 22], the correlations between PASI score and the serum levels of some cytokines suggest that serum cytokine levels could be used as an objective parameter to reflect the disease activity of psoriasis.

The cathelicidin LL-37 is overexpressed in skin lesions [10, 23] and has recently been identified as a critical factor for the activation of an autoinflammatory cascade in psoriasis [8]. In the present study, the serum LL-37 levels were elevated in patients with psoriasis compared with healthy controls but the levels were not correlated with the PASI score. This finding is consistent with previous reports that serum LL-37 is not related to psoriasis severity [12, 24]. One possible explanation for the lack of an association between PASI and serum LL-37 is that, unlike the LL-37 found in lesional skin, the serum type represents the sum total produced by LL-37-producing cells in the intestine, airways, lymph nodes, and bone marrow. Thus, the serum LL-37 level could be affected by multiple organs' immune activities as well as many other factors such as vitamin D levels or ultraviolet and microbial exposure in the environment. In addition, LL-37 can play both a regulatory and a provocative role in immune responses [11, 12, 25]. It may also be a modulator acting to balance the levels of pro- and anti-inflammatory cytokines [26], and hence it may not be positively correlated with disease activity.

As with the cytokines, the serum level of LL-37 did not differ significantly between the guttate and plaque morphologic types, which suggests that serum LL-37 is not involved in the establishment of a particular psoriasis phenotype.

This study found that serum LL-37 levels were significantly correlated with serum IFN-*γ* and IL-22 levels. The circulating LL-37 appeared to affect both INF-*γ* and IL-22, both of which are key cytokines in the Th1 and Th17 pathways involved in psoriasis. Sophisticated interactions between the cathelicidin and these 2 cytokines might exist [26–29], but no clear mechanism has yet been verified. Further investigation is necessary to determine the clinical significance and possible mechanism of this putative interaction.

In summary, the circulating levels of inflammatory cytokines and LL-37 do not appear to differ significantly between the guttate and plaque types of psoriasis. Serum levels of inflammatory cytokines appear to reflect disease activity rather than the morphological phenotype. Serum LL-37 is elevated in psoriasis but is not associated with either the morphological phenotype or the disease activity of psoriasis. Further studies are still needed to elucidate the relationship between serum LL-37 and cytokines in the pathogenesis of psoriasis and its clinical implications.

## Figures and Tables

**Table 1:** Demographics of healthy controls and patients with 1 of 2 types of psoriasis.

	Healthy controls	Psoriasis patient	Guttate psoriasis	Plaque psoriasis
Number	12	74	32	42
Age, years	32.6 ± 10.0	36.5 ± 14.2 (15~77)	37.7 ± 14.8	35.6 ± 13.9
Duration of psoriasis, years	N/A	6.2 ± 7.3	6.1 ± 7.5	6.2 ± 7.3
PASI score	N/A	7.9 ± 3.9	7.2 ± 3.4	9.1 ± 4.1

PASI: Psoriasis Area and Severity Index; N/A: not applicable.

Except where indicated otherwise, data are mean ± SD values.

**Table 2:** Statistical analysis results of serum levels of cytokines and LL-37 in patients with psoriasis and healthy controls.

	Patients (*n* = 74) Median (IQR), mean	Healthy controls (*n* = 12) Median (IQR), mean	*P* ^a^
IL-1RA (pg/mL)	2.26 (0.00–7.18), 4.40	0.00 (0.00–1.62), 1.47	0.020∗
IL-2 (pg/mL)	0.81 (0.59–1.25), 1.05	0.00 (0.00–0.92), 0.84	0.007∗
IL-12 (pg/mL)	2.71 (0.00–4.80), 3.23	0.00 (0.00–1.36), 1.33	0.071
IL-17A (pg/mL)	0.31 (0.00–1.37), 1.59	0.00 (0.00–2.75), 1.52	0.936
IL-22 (pg/mL)	0.11 (0.09–0.18), 0.15	0.10 (0.10–0.13), 0.11	0.625
IL-23 (pg/mL)	1.57 (1.44–2.00), 1.99	0.95 (0.45–1.24), 1.02	<0.001∗
IFN-*γ* (pg/mL)	2.94 (2.46–5.18), 4.53	0.50 (0.00–2.06), 1.34	<0.001∗
LL-37 (ng/mL)	11.78 (9.75–16.90), 13.46	8.73 (7.73–11.60), 9.43	0.011∗

IL: interleukin; IL-1RA: IL-1 receptor antagonist; IFN: interferon; IQR: interquartile range.

^a^Mann-Whitney *U* test between healthy controls and patients with psoriasis.

∗denotes statistically significant difference at *P* < 0.05.

**Table 3:** Statistical analysis results of levels of specific serum cytokines and LL-37 in patients with guttate and plaque psoriasis.

	Guttate psoriasis (*n* = 32) median (IQR), mean	Plaque psoriasis (*n* = 42) median (IQR), mean	*P* ^a^
IL-1RA (pg/mL)	2.75 (1.12–7.18), 4.84	1.20 (0.00–5.21), 4.06	0.229
IL-2 (pg/mL)	0.81 (0.70–1.31), 0.11	0.84 (0.59–1.14), 1.00	0.558
IL-12 (pg/mL)	0.00 (0.00–4.80), 2.75	2.71 (0.00–5.91), 3.61	0.320
IL-17A (pg/mL)	0.00 (0.00–1.23), 1.31	0.68 (0.00–1.54), 1.80	0.580
IL-22 (pg/mL)	0.10 (0.09–0.14), 0.14	0.11 (0.09–0.20), 0.15	0.443
IL-23 (pg/mL)	1.57 (1.44–1.86), 2.01	1.58 (1.44–2.03), 1.97	0.756
IFN-*γ* (pg/mL)	2.90 (2.62–5.00), 4.64	3.02 (2.46–5.18), 4.45	0.768
LL-37 (ng/mL)	10.95 (8.96–16.94), 13.20	12.56 (10.60–16.90), 13.65	0.204

^a^Mann-Whitney *U* test between patients with guttate psoriasis and plaque psoriasis.

**Table 4:** Correlations between disease severity (PASI score) and serum levels of cytokines and LL-37 in all patients with psoriasis.

	Statistical parameters	IL-1RA	IL-2	IL-12	IL-17A	IL-22	IL-23	IFN-*γ*	LL-37
PASI	*r*	0.21	0.16	0.30	0.21	0.26	0.10	0.23	0.08
	*P*	0.07	0.16	0.01∗	0.07	0.02∗	0.42	0.048∗	0.50

*r*: Pearson correlation coefficient.

∗denotes statistically significant difference at *P* < 0.05.

**Table 5:** Correlations between serum levels of LL-37 and cytokines in patients with psoriasis.

LL-37	*r*	*P*
IL-1RA	0.16	0.18
IL-2	0.04	0.75
IL-12	0.03	0.81
IL-17A	0.20	0.09
IL-22	0.24	<0.05∗
IL-23	0.17	0.15
IFN-*γ*	0.31	<0.01∗

*r*: Pearson correlation coefficient.

∗denotes statistically significant difference at *P* < 0.05.
